# Site-Specific Volumetric Skeletal Changes in Women with a Distal Forearm Fracture

**DOI:** 10.1155/2021/1578543

**Published:** 2021-09-30

**Authors:** Axel Wihlborg, Karin Bergström, Ingrid Bergström, Paul Gerdhem

**Affiliations:** ^1^Department of Clinical Science, Intervention and Technology, Karolinska Institutet, Department of Orthopedics, K54, Karolinska University Hospital, Huddinge, SE-141 86 Stockholm, Sweden; ^2^Karolinska Institutet, Department of Endocrinology, Metabolism & Diabetes, Karolinska University Hospital, Huddinge, SE-141 86 Stockholm, Sweden; ^3^Department of Clinical Science, Intervention and Technology, Karolinska Institutet, Department of Endocrinology, Metabolism & Diabetes, Karolinska University Hospital, Huddinge, SE-141 86 Stockholm, Sweden

## Abstract

**Purpose:**

To assess site-specific volumetric bone and muscle changes, as well as demographic and biochemical changes, in postmenopausal women with a low-energy distal forearm fracture.

**Methods:**

In a cross-sectional case-control study, postmenopausal women with a distal forearm fracture were compared with age- and gender-matched controls. In total, 203 postmenopausal women (104 cases and 99 controls), with a mean age of 65 years, were included. Measurements included peripheral quantitative computed tomography (pQCT) and dual-energy X-ray absorptiometry (DXA) as well as blood sampling and questionnaires.

**Results:**

Forearm trabecular volumetric BMD and total BMD assessed with pQCT were significantly lower in fracture cases compared to controls (*p* < 0.001). Significantly higher cross-sectional area, lower cortical BMD, and lower cortical thickness were seen in women with fracture (*p* < 0.033, *p* < 0.001, and *p* < 0.001, respectively). Postmenopausal women with fracture had significantly lower hip and spine areal BMD assessed with DXA (*p* < 0.001). Activity level was higher and a history of falling was more frequent in women with fracture (*p* < 0.019 and *p* < 0.001, respectively). Vertebral fracture was observed in 24 women (22%) with a distal forearm fracture. Muscle area, muscle density, PTH, and 25OHD did not differ between fracture cases and controls.

**Conclusion:**

A distal forearm fracture was associated with site-specific and central bone changes. Postmenopausal women with fracture had a larger bone area in combination with a thinner cortex and lower site-specific total BMD. In addition, women with fracture had a higher activity level, an increased occurrence of previous fall accidents, and a high prevalence of vertebral fractures. Forearm muscle composition, PTH, and 25OHD were not associated with forearm fracture. Fracture preventive measures following a low-energy distal forearm fracture seem beneficial.

## 1. Introduction

Distal forearm fracture is one of the most common fractures in postmenopausal women, associated with an increased risk of subsequent fracture and to some extent osteoporosis [1–3]. Geometrical changes and bone mass reductions in both trabecular and cortical bone compartments of the radial bone have been suggested as contributing factors for fracture [4–6]. However, conclusive evidence in clinical settings is sparse [7–11].

Areal bone mineral density (aBMD) of the hip measured by dual-energy X-ray absorptiometry (DXA) and site-specific aBMD of the forearm have been shown to correlate with an increased risk of forearm fracture [8, 10, 12–14]. However, aBMD incompletely explains aspects of bone strength potentially related to geometric properties and composition of the cortical and trabecular compartments [4]. A relative difference between trabecular and cortical impairment, as well as geometrical changes, such as changes in bone size, may affect the resistance to fracture. These properties can be evaluated by peripheral quantitative computed tomography (pQCT), a three-dimensional technique that determines true volumetric bone mineral density (BMD). pQCT allows for a separate evaluation of trabecular and cortical compartments and provides information on geometric properties such as cortical thickness and bone size. Recent findings have suggested correlations between pQCT variables with forearm fracture risk [7–11].

Geometric changes with increased bone size have previously been observed in women during ageing [4, 15–17]. It has been suggested that these geometric alterations, which are favorable in terms of structural strength, compensate for age-related reductions in bone mass [4, 15–17]. Although not previously described, it is reasonable to assume similar geometric alterations in women with a distal forearm fracture and decreased bone mass independent of age.

In addition to skeletal variables, volumetric muscle density and area may be estimated by pQCT [4]. According to the Mechanostat theory, bone remodulation is in close affinity with muscular loading, and site-specific muscle composition and strength may influence radial bone strength [18]. Decreased muscle density as well as grip strength has been observed in women with a distal forearm fracture [7], but have not been studied in larger cohorts.

Physical ability is also likely to affect the risk of fracture. We have previously shown a correlation between history of falls and subsequent increased risk of distal forearm fracture [19], and it has been suggested that a forearm fracture is more likely to occur in postmenopausal women with somewhat higher activity level in combination with an increased tendency to fall [13, 20].

We hypothesized that women with a distal forearm fracture would exhibit decrements of both trabecular and cortical bone mass and that these reductions would be accompanied by geometric alterations of the bone. In addition, we hypothesized changes in physical activity and in the tendency to fall. To explore these hypotheses, we invited women with a distal forearm fracture to participate in a case-control study.

## 2. Materials and Methods

### 2.1. Study Population

**Figure 1:**
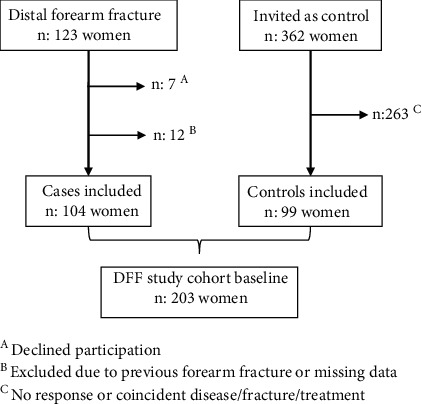
Recruitment of women with a forearm fracture and age-matched controls in the Distal Forearm Fracture (DFF) study.

This study consists of postmenopausal women participating in the Distal Forearm Fracture (DFF) study. The DFF study was conducted at Karolinska University Hospital in Stockholm, Sweden. Postmenopausal women presenting with a low-energy distal forearm fracture at the orthopedic emergency department, from April 2010 to January 2015, were invited to attend the study. Of the 123 women who initially consented to participate, 7 declined to come to the research department, 9 were excluded due to a previous contralateral fracture, and 3 had missing data, leaving 104 women, with a mean age of 64, in the study. Age- and gender-matched controls from the same geographic area (Huddinge, Sweden) were selected at random through the population register. Invitations were sent by mail, with one reminder, to 362 women in total. Controls were excluded in case of a history of osteoporosis-related fracture (including a distal forearm fracture), known bone remodeling disease, or antiresorptive, estrogen, or oral corticosteroid treatment. In total, 99 controls with a mean age of 65 years were included (Figure 1).

### 2.2. Measurements

The examination protocol was identical for cases and controls, and an identical self-assessment questionnaire was used in both cases and controls. All distal forearm fractures were radiologically confirmed and resulting from low-energy trauma. In addition to the examination protocol, women with a forearm fracture were clinically evaluated and referred to spine radiograph in the presence of significant height loss, kyphosis, or back pain. An independent radiologist assessed the spine radiograph.

Volumetric properties of the forearm were assessed by peripheral quantitative computed tomography (pQCT) with Stratec XTC-2000 (Stratec Medizintechnik, Pforzheim, Germany). The in-house coefficient of variation (CV) was 0.27% using the forearm phantom. Version 6.2 of the manufacturer software was used. Measurements were performed at the distal (4%) and shaft (66%) site of the radial bone with 0.5 mm voxel size, 2.3 mm slice thickness, and 20 mm/s scanning speed. Contour mode 1 was used with a density threshold of 180 mg/cm^3^ at the distal (4%) site and 280 mg/cm^3^ at the shaft (66%) site to separate the outer edge of bone from soft tissue. Trabecular bone was distinguished by 45% of the area at the distal site (peel mode 1). Cortical bone was distinguished by cortical mode 1 with 711 mg/cm^3^ inner threshold at the shaft site. Muscle tissue was separated by contour mode 3 with a threshold of 40 mg/cm^3^ at the shaft site. Trabecular bone properties were assessed at the 4% site by trabecular BMD, total bone mineral content (BMC), and total BMD. Cortical bone properties were assessed at the 66% site by total BMC and cortical BMD. Bone geometric properties were assessed by cross-sectional area (CSA) at the 4% site as well as cortical thickness, periosteal and endosteal circumference, and CSA at the 66% site. Bone strength was assessed by stress-strain index (SSI) in respect to torsion load (SSIp) and bending load (SSIx and SSIy) at the 66% site. Cortical variables were retrieved at the shaft site due to pQCT-associated measurement uncertainty of cortical variables at the distal site. Cortical bone properties at the shaft site were presumed to reflect cortical bone properties throughout the bone. Muscle properties were assessed as muscle area and density.

Areal bone mineral density (aBMD) was measured at the hip, lumbar spine, and distal radius with GE Lunar iDXA (GE Medical Systems, Chalfont St. Giles, UK). Site-specific aBMD of the forearm was assessed at the ultradistal site (UDR) and at the 33% site. Coefficient of variation (CV) was 1.5% for the spine phantom provided by the manufacturer. BMD measurements were performed as per manufacturer's instructions including calibrations with a phantom.

DXA and pQCT were performed at the nonfractured forearm with matching number of left/right side in the controls. The left arm was examined in 49 of the women with fracture and in 56 of the controls.

Blood samples were drawn at the research facility, and serum were frozen and stored at −80°C. Parathyroid hormone was analyzed as intact PTH on Modular Analytics E170 (Roche Diagnostics Ltd.) using electrochemiluminescence immunoassay (ECLIA) with intra- and interassay CV ranging between 1.1-2.0% and 2.8–3.4%, respectively. 25-Hydroxy vitamin D3 (25OHD) was analyzed on API 4000 LC-MS/MS (Sciex), with assay CV ranging between 4.0 and 6.0%. Calcium was analyzed on Cobas c502 (Roche Diagnostics Ltd.) by the NM-BAPTA method, with assay CV ranging between 0.6 and 0.5%. Serum calcium was corrected for serum albumin by the following formula: calcium + 0.01 × (39-albumin).

The study was approved by the Regional Ethical Board in Stockholm (2009/913–31). Informed consent was obtained from all participants. Fractures and detected diseases were treated in accordance with clinical routine.

### 2.3. Statistical Analysis

The independent *t*-test was used to determine mean difference of continuous variables, and Pearson's chi-squared test or Fisher's exact test was applied for categorical variables. Comparisons of site-specific pQCT and DXA variables between fracture and control groups were made with independent *t*-tests. Analysis of covariance (ANCOVA) was used in statistical adjustment for possible confounding variables; age, body mass index (BMI), 25OHD, bisphosphonate use, and femoral neck aBMD by DXA. Continuous data were visually controlled for normal distribution, and logarithmized variables were used in the statistical analyses in case of nonnormality. *p* < 0.05 was considered significant. SPSS Statistics version 20 was used for all statistical analysis.

To identify a 10% difference in bone density (corresponding to 20 mg/cm^3^ total BMD) with a standard deviation of 22.5% (corresponding to 45 mg/cm^3^) with alpha 0.05 and 80% power, a case to control ratio of 1 : 1, 81 cases and 81 controls, was needed.

## 3. Results

Descriptive characteristics of the study population are shown in Tables 1 and 2. Women with a distal forearm fracture had lower central BMD (hip and spine), more frequently reported a history of falling, and reported more time spent walking. There were no differences in age, BMI, smoking habits, alcohol consumption, disease, estrogen use, cortisone use, bisphosphonate use, mean PTH, 25OHD, or serum calcium between cases and controls (Tables 1 and 2).

The presence of osteoporosis defined as *T*-score ≤−2.5 was more frequent among women with a distal forearm fracture (Table 1). Out of the 104 women with fracture, 83 performed a spine radiograph and 24 women (22%) were subsequently diagnosed with vertebral fracture.

Forearm bone and muscle characteristics are shown in Table 3. Total volumetric BMC and BMD as well as trabecular BMD at the 4% site were lower in women with fracture. In addition, volumetric cortical estimates (cortical BMD and total BMC at the 66% site) were lower in women with fracture. Areal BMD determined by DXA at the ultradistal site (UDR) and at the 33% site were lower in women with fracture.

Cross-sectional area (CSA) at the 4% site was greater, while cortical thickness was lower in women with fracture. No difference in periosteal circumference was observed, while endosteal circumference was greater in women with fracture (Table 3).

No differences in stress-strain index (SSI), muscle density, or muscle area were observed. All changes remained significant after adjustment for possible confounding variables in ANCOVA analyses, except aBMD at the 33% site (Table 3).

## 4. Discussion

In this cross-sectional case-control study, we demonstrated volumetric skeletal changes of the forearm in women with a distal forearm fracture when compared to age-matched controls. Osteoporosis was more common among women with a distal forearm fracture, who also reported an increased physical activity and more frequent falls. Forearm muscle composition, PTH, and 25OHD were not associated with distal forearm fracture.

The decreased trabecular and cortical density in postmenopausal women with fracture indicates decreased structural strength in terms of withstanding compressional load at the metaphyseal end of the bone. However, the trabecular deterioration appeared to be more pronounced based on a larger observed difference. Our findings are strengthened by previous observations with pQCT [7, 11] and high-resolution pQCT [8–10], although these studies are not comparable in terms of cohort size.

In addition, changes in geometric parameters were observed. Among postmenopausal women with fracture, the cross-sectional area of the distal radial bone was greater, while cortical thickness was lower. This suggests that women prone to distal forearm fractures exhibit a radial bone with increased width and thinner cortex. Similar geometric changes have previously been observed in women during ageing, and it has been proposed to be a compensatory mechanism by which structural strength is maintained with age-related reductions in bone mass [4, 15–17]. These geometric changes have however not previously been observed in women with fracture [8–10]. The increased cross-sectional area at the distal site in this study may be an effect of decreased bone mass, but apparently insufficient in terms of maintaining structural strength.

Neither the cross-sectional area at the diaphyseal 66% site nor the stress-strain index derived from measures at the same site differed between postmenopausal women with and without fracture. These findings suggest less pronounced differences at the diaphyseal site, which primarily consists of cortical bone and is located at a greater distance from the site of fracture.

Areal BMD by DXA at the ultradistal site was reduced in postmenopausal women with fracture, which were in line with the volumetric density findings by pQCT. Decreased areal BMD has previously been shown in women with fracture [8, 10, 12, 14] and may serve as a reasonable estimator of structural strength in terms of withstanding compressional load.

Important findings in this study were that postmenopausal women with fracture had a greater activity level and an increased occurrence of previous fall accidents, suggesting that factors other than bone mineral density and geometry are of importance in distal forearm fracture predisposition [13, 19, 20]. It is well known that postural balance deteriorates during menopause due to estrogen loss [21], and this might be one of several explanations for the increased risk of falling. Fall-preventive interventions with exercise and balance training have shown to reduce the risk of falling also in the elderly population [22, 23] and might therefore be advised following a distal forearm fracture.

We were not able to demonstrate changes in muscle area or muscle density of the forearm, although decreased muscle density as well as grip strength has previously been suggested in women with a distal forearm fracture [7]. Other factors, including history of falls, are likely to be more important than forearm muscle function for fracture risk [19].

The categorization of low-energy distal forearm fractures as fragility fractures has been a subject of debate in specialized bone societies, as has the clinical evaluation in terms of osteoporosis following a distal forearm fracture [24]. Nevertheless, the risk of future osteoporosis-related fractures seems to be increased in this group of women [24]. The decreased central and peripheral BMD, the increased occurrence of falls, and the high prevalence of vertebral fractures [25] suggest attention towards fracture risk assessment after a low-energy distal forearm fracture [13, 19, 20, 26].

This study has many advantages. It is a large case-control cohort with a sufficient sample size according to power analysis, and the cohort size is larger than previous comparable studies. The cases as well as the age- and gender-matched controls underwent an identical examination protocol.

There are some limitations associated with the study. A slow recruitment rate was seen, caused by unwanted interruptions of inclusion due to administrative reasons within the hospital system. We do however not believe this have led to a recruitment bias. Direct measurements of bone strength and muscular load by biomechanical tests were not available.

In summary, this study revealed several significant differences between postmenopausal women with a distal forearm fracture compared to age-matched controls. Women with a distal forearm fracture exhibited reductions in both cortical and trabecular bone compartments, combined with altered geometric composition of the radial bone in the forearm. Furthermore, postmenopausal women with fracture had significantly decreased central aBMD in combination with greater activity level and increased history of falls. These findings suggest that fall and fracture preventive measures could be beneficial following a low-energy distal forearm fracture in postmenopausal women.

## Figures and Tables

**Table 1:** Descriptive statistics of women with a distal forearm fracture and age-matched controls.

	Control	Fracture	Significance
	*n* = 99	*n* = 104
	Mean (SD)	Mean (SD)	*p* value^*∗*^
Age (years)	63.9 (7.7)	65.5 (8.9)	0.18
Body height (m)	1.64 (0.06)	1.64 (0.06)	0.84
Body weight (kg)	70.3 (12.8)	68.8 (11.0)	0.37
BMI (kg/m^2^)	26 (5)	26 (4)	0.50
Serum calcium (mmol/L)	2.38 (0.12)	2.37 (0.09)	0.30
Parathyroid hormone (pmol/L)	4.9 (2.3)	4.7 (2.2)	0.51
PHPT^a^	4 (4)^*∗∗*^	4 (4)^*∗∗*^	1.0
25OHD (nmol/L)	59 (21)	64 (24)	0.13
Vitamin D insufficiency^b^	31 (33)^*∗∗*^	28 (27)^*∗∗*^	0.38
DXA			
aBMD femoral neck (g/cm^2^)	0.871 (0.122)	0.810 (0.104)	<0.001
*T*-score femoral neck	−1.2 (0.9)	−1.6 (0.7)	<0.001
aBMD L1–L4 (g/cm^2^)	1.114 (0.175)	0.995 (0.130)	<0.001
*T*-score L1–L4	−0.6 (1.4)	−1.5 (1.0)	<0.001
Osteoporosis^c^	17 (18)^*∗∗*^	39 (38)^*∗∗*^	0.001

Continuous variables are described as means and standard deviations (SD). Dichotomous variables are described as frequencies and percentages. BMI, body mass index; aBMD, areal bone mineral density by DXA.^*∗*^*T*-test. PHPT, vitamin D insufficiency and osteoporosis: Pearson's chi-squared test or Fischer's exact test ^*∗∗*^Number (%). ^a^Primary hyperparathyroidism defined as PTH >9.6 pmol/L combined with calcium >2.5 mmol/L. ^b^25OHD <50 nmol/L. ^c^*T*-score ≤−2.5 in the hip or spine.

**Table 2:** Descriptive statistics of women with a distal forearm fracture and age-matched controls, based on self-reported questionnaire.

	Control	Fracture	Significance
	*n* = 99	*n* = 104
	Number (%)	Number (%)	*p* value^*∗*^
Completed questionnaire	97 (98)	99 (95)	0.28
Smoking	8 (8)	11 (11)	0.50
Alcohol use (units/day)	0.54 (0.75)^*∗∗*^	0.51 (0.71)^*∗∗*^	0.76
Walking one hour or more/day	29 (31)	46 (48)	0.019
History of a fall^a^	31 (32)	69 (70)	<0.001
History of multiple falls^b^	15 (15)	34 (34)	0.002
History of osteoporosis-related fracture	0	11 (6)	0.001

*Disease*			
Kidney disease	0	2 (2)	0.50
Rheumatic disease	5 (5)	8 (8)	0.41
Diabetes mellitus	6 (6)	4 (4)	0.54
Cancer	11 (11)	10 (10)	0.78

*Medication*			
Estrogen use	0	3 (3)	0.25
Cortisone use	0	2 (2)	0.50
Vitamin D supplementation	1 (1)	7 (7)	0.06
Bisphosphonate use	0	2 (2)	0.50

Dichotomous variables are described as frequencies and percentages of those with completed questionnaires. Alcohol use is described as a continuous variable with mean and standard deviation (SD).^*∗*^Pearson's chi-squared test or Fischer's exact test. Alcohol use: *T*-test. ^*∗∗*^Mean (SD). ^a^Reported at least one fall within 1 year prior to investigation point. ^b^Reported two or more falls within 1 year prior to investigation point.

**Table 3:** Site-specific bone and muscle variables in the DFF cohort.

	Control	Fracture	Mean difference (CI)	Difference %	Significance	Significance
	*n* = 99	*n* = 104			*T*-test	ANCOVA
	Mean (SD)	Mean (SD)			*p* value	*p* value
BMC total 4% (mg/mm)	97.3 (19.5)	84.8 (12.9)	−12.5 (−17.0 to −7.9)	13	<0.001	<0.001
BMD total 4% (mg/cm^3^)	309.2 (63.3)	256.8 (47.0)	−52.6 (−67.9 to 37.2)	17	<0.001	<0.001
BMD trabecular 4% (mg/cm^3^)	167.5 (43.3)	125.1 (31.7)	−42.4 (−52.9 to −31.9)	25	<0.001	<0.001
BMC total 66% (mg/mm)	89.8 (16.5)	81.4 (14.7)	−8.5 (−12.8 to −4.1)	9	<0.001	0.027
BMD cortical 66% (mg/cm^3^)	1120.8 (43.8)	1095.7 (55.7)	−25.1 (−39.0 to −11.2)	2	<0.001	0.007
aBMD UDR (g/cm^2^)	0.392 (0.079)	0.327 (0.060)	− 0.065 (−0.085 to −0.046)	17	<0.001	<0.001
aBMD 33% of radius (g/cm^2^)	0.782 (0.104)	0.731 (0.114)	−0.051 (−0.082 to −0.021)	7	0.001	0.18

CSA 4% (mm^2^)	319.8 (56.3)	336.4 (53.5)	16.6 (1.4 to 31.8)	5	0.033	0.037
CSA 66% (mm^2^)	130.3 (23.2)	134.3 (26.8)	4.0 (−3.0 to 10.9)	3	0.26	0.38
Cortical thickness (mm)	1.996 (0.401)	1.726 (0.409)	− 0.270 (− 0.382 to −0.158)	14	<0.001	<0.001
Periosteal circumference (mm)	40.3 (3.5)	40.9 (3.8)	0.6 (−0.4 to 1.6)	2	0.25	0.36
Endosteal circumference (mm)	27.8 (4.5)	30.1 (5.3)	2.3 (0.9 to 3.6)	8	0.001	0.009

SSIp (mm^3^)	264.1 (57.8)	253.0 (51.5)	−11.1 (−26.2 to 4.0)	4.2	0.15	0.30
SSIx (mm^3^)	141.9 (33.4)	134.6 (29.3)	−7.4 (−16.0 to 1.3)	5.2	0.12	0.25
SSIy (mm^3^)	150.6 (36.1)	142.1 (31.2)	−8.5 (−17.8 to 0.8)	5.6	0.10	0.22

Muscle area (mm^2^)	2630.4 (355.6)	2646.8 (370.8)	16.4 (−84.5 to 117.2)	1	0.75	0.24^*∗*^
Muscle density (mg/cm^3^)	74.1 (1.9)	73.7 (2.1)	0.5 (−1.0 to 0.1)	1	0.12	0.17^*∗*^

Values are depicted as mean with standard deviation (SD) and mean difference with confidence interval (CI). BMC and BMD: bone mineral content and density assessed by pQCT; aBMD: areal bone mineral density by DXA; UDR: ultradistal radius; CSA: cross-sectional area; ANCOVA: analysis of covariance (covariates: age, BMI, 25OHD, and femoral neck aBMD) ^*∗*^Bisphosphonate use and femoral neck aBMD not included as covariates.

## Data Availability

The data used to support the findings of this study are available from the corresponding author upon request.
